# Supplementary material for: Bacillus simplex treatment promotes soybean defence against soybean cyst nematodes: A metabolomics study using GC-MS
Source: PLoS One. 2020 Aug 6;15(8):e0237194. doi: 10.1371/journal.pone.0237194 (PMC7410315; doi:10.1371/journal.pone.0237194)
Supplement: Attachment — Submitted filename: Response to Reviewers.docx [file pone.0237194.s005.docx]

Response to Reviewers

Dear Editors and Reviewers:

Thank you for your letter and for the reviewers’s comments concerning our manuscript entitled “Bacillus simplex treatment promotes soybean defence against soybean cyst nematodes: A metabolomics study using GC-MS”(PONE-D-20-07320). Those comments are all valuable and very helpful for revising and improving our paper, as well as the important guiding significance to our researches. We have studied comments carefully and have made correction which we hope meet with approval. Revised portion are marked in red in the paper. The main corrections in the paper and the responds to the editors and reviewer’s comments are as flowing:

**Responds to the editor’s comments:**

1. We have found the professional English editing for this manuscript to improve its language clarity. And we have different treatment manuscript labeled followed the 'Response to Reviewers', 'Revised Manuscript with Track Changes' and 'Manuscript'.
2. We have deposited our laboratory protocols in protocols.io.

DOI: dx.doi.org/10.17504/protocols.io.bhbpj2mn

3. We have added the first author’s ORCID.
4. We have also arranged professional English editing for this manuscript to improve its language clarity.
5. We have uploaded our minimal data to a public repository Figshare.

Supporting information file 1 DOI: 10.6084/m9.figshare.12510803

Supporting information file 2 DOI: 10.6084/m9.figshare.12510809

Supporting information file 3 DOI: 10.6084/m9.figshare.12510824

Supporting information file 4 DOI: 10.6084/m9.figshare.12510842

We have added the supporting information files at end of our manuscript.

**Responds to the reviewer’s comments:**

Reviewer 2:

1.	Response to comment: L. 47-50: the objective is quite general. It would be good to add hypotheses of this study.

**Reply: We added the hypotheses of this study that “We hypothesized that bacteria could improve soybean resistance to nematode infection by inducing the soybean plant roots to produce substances with nematode killing activity or inhibiting the nematode’s growth and development.” in L.53-56.**

2.	Response to comment: L. 59: not clear of how the bacteria inoculum were coated to the seeds.

**Reply: We explain the methods how the bacteria inoculum were coated to the seeds in L.66-67 “Subsequently, the seeds were washed with 70% ethanol and 1% of the seeds were coated with Sneb545.”**

3.	Response to comment: L. 67: not clear of the seedling age or growth stage at the time of inoculation with SCN.

**Reply: L.77-78 When two true leaves were grown from soybean, the root of soybean was inoculated with soybean cyst nematode.**

4.	Response to comment: L. 70: plants were assessed at 5 and 10 days after inoculation: not sure if such short period was enough for observing interactions between the bacteria and SCN

**Reply: L79: After the previous observation on the development of nematode in soybean root under the microscope, we found that there were four larvae at 10 days after nematode infected soybean, so although the difference was only 5 days, the development state of nematode in these two time points was obviously different.**

5.	Response to comment: L. 81: samples of roots?

**Reply: Yes. The sample is root tissue of the soybean.**

6.	Response to comment: L. 74: ‘Liaodou15’ here, but ‘Liao15’ in L. 58. Use one and be consistent throughout the manuscript

**Reply: We have changed the L.84‘Liaodou15’ into‘Liao15’.**

7.	Response to comment: L. 77-78: last sentence on data analysis can be removed to the “Data analysis” section

**Reply: We removed the L.77-78 last sentence on data analysis to the “Statistical analysis” section in L.120-121 “The nematode infection and mortality data were examined using Student’s t-test in SPSS 17.0 (IBM, USA) and a P-value of < 0.05 was considered statistically significant.”**

8.	Response to comment: L. 107-114: put this section last, i.e. before the ‘Results’ section to include all acquired data in this study in addition to the spectrometry data, such as ANOVA for nematode numbers etc.

**Reply: We put this section ‘Statistical analysis’ before the‘Results’ section in L.120-128.**

9.	Response to comment: L. 129: significant level? The statistical analysis was not mentioned in the M&M section.

**Reply: We added the statistical analysis in the ‘Statistical analysis’ section in L.121-123.**

10.	Response to comment: L. 130-131: delete this sentence and move it to Discussion section.

**Reply: We delete this sentence and move it to Discussion section in L.162-163.**
