# Supplementary material for: Bacillus simplex treatment promotes soybean defence against soybean cyst nematodes: A metabolomics study using GC-MS
Source: PLoS One. 2020 Aug 6;15(8):e0237194. doi: 10.1371/journal.pone.0237194 (PMC7410315; doi:10.1371/journal.pone.0237194)
Supplement: Attachment — Submitted filename: Response to Reviewers.docx [file pone.0237194.s006.docx]

Response to Reviewers

Dear Editors and Reviewers:

Thank you for your letter and for the reviewers’s comments concerning our manuscript entitled “Bacillus simplex treatment promotes soybean defence against soybean cyst nematodes: A metabolomics study using GC-MS”(PONE-D-20-07320). Those comments are all valuable and very helpful for revising and improving our paper, as well as the important guiding significance to our researches. We have studied comments carefully and have made correction which we hope meet with approval. Revised portion are marked in red in the paper. The main corrections in the paper and the responds to the editors and reviewer’s comments are as flowing:

**Responds to the reviewer’s comments:**

Reviewer 2:

1.	Response to comment: Higher resolution/better quality of figures are required for the publications.

**Reply: We enhanced the resolution to 300*300 of the figures .**

2.	Response to comment: change "Soybean has a high protein and oil content" to "Soybean seeds contain high protein and oil content".

**Reply: We have changed the sentence to “Soybean seeds contain high protein and oil content” in L.33**

3.	Response to comment: L. 34 "which place high importance on its production yield" is not clear.

**Reply: We have changed the sentence to “This places a high importance on efficient production of soybean with a high yield” in L. 34.**

4. Response to comment: L. 36 "can hinder its growth and production": not clear of "its" - just use "soybean", and can be changed to "can hinder the growth and production of soybean".

**Reply: We have changed the sentence to “can hinder the growth and production of soybean” in L.37.**

5. Response to comment: L. 39-40: "Plant growth-promoting rhizobacteria (PGPR) colonises the rhizosphere of" should use "colonise".

**Reply: We have changed the word to “colonise” in L.41.**

6. Response to comment: L. 42-44: replace "Our past studies have discovered the Sneb545 strain of Bacillus simplex that can enhance soybean resistance to SCN" with "Our past studies have discovered that the Sneb545 strain of Bacillus simplex enhanced soybean resistance to SCN"

**Reply: We have changed the sentence to "Our past studies have discovered that the Sneb545 strain of Bacillus simplex enhanced soybean resistance to SCN" in L.43-45.**

**7. We also modified the other language problems in the ‘Revised Manuscript with Track Changges’**
